# Deficits in Endogenous Adenosine Formation by Ecto-5′-Nucleotidase/CD73 Impair Neuromuscular Transmission and Immune Competence in Experimental Autoimmune Myasthenia Gravis

**DOI:** 10.1155/2015/460610

**Published:** 2015-01-27

**Authors:** Laura Oliveira, Alexandra Correia, Ana Cristina Costa, Sónia Guerra-Gomes, Fátima Ferreirinha, Maria Teresa Magalhães-Cardoso, Manuel Vilanova, Paulo Correia-de-Sá

**Affiliations:** ^1^Laboratório de Farmacologia e Neurobiologia, UMIB and MedInUP, Instituto de Ciências Biomédicas de Abel Salazar (ICBAS), Universidade do Porto (UP), Rua de Jorge Viterbo Ferreira No. 228, Edificio 2 Piso 4, 4050-313 Porto, Portugal; ^2^Laboratório de Imunologia, Departamento de Imunofisiologia e Farmacologia, Instituto de Ciências Biomédicas de Abel Salazar (ICBAS), Universidade do Porto (UP), Rua de Jorge Viterbo Ferreira No. 228, Edificio 2 Piso 4, 4050-313 Porto, Portugal; ^3^IBMC-Instituto de Biologia Celular e Molecular, Universidade do Porto, Rua do Campo Alegre No. 823, 4150-180 Porto, Portugal

## Abstract

AMP dephosphorylation via ecto-5′-nucleotidase/CD73 is the rate limiting step to generate extracellular adenosine (ADO) from released adenine nucleotides. ADO, via A_2A_ receptors (A_2A_Rs), is a potent modulator of neuromuscular and immunological responses. The pivotal role of ecto-5′-nucleotidase/CD73, in controlling extracellular ADO formation, prompted us to investigate its role in a rat model of experimental autoimmune myasthenia gravis (EAMG). Results show that CD4^+^CD25^+^FoxP3^+^ regulatory T cells express lower amounts of ecto-5′-nucleotidase/CD73 as compared to controls. Reduction of endogenous ADO formation might explain why proliferation of CD4^+^ T cells failed upon blocking A_2A_ receptors activation with ZM241385 or adenosine deaminase in EAMG animals. Deficits in ADO also contribute to neuromuscular transmission failure in EAMG rats. Rehabilitation of A_2A_R-mediated immune suppression and facilitation of transmitter release were observed by incubating the cells with the nucleoside precursor, AMP. These findings, together with the characteristic increase in serum adenosine deaminase activity of MG patients, strengthen our hypothesis that the adenosinergic pathway may be dysfunctional in EAMG. Given that endogenous ADO formation is balanced by ecto-5′-nucleotidase/CD73 activity and that A_2A_Rs exert a dual role to restore use-dependent neurocompetence and immune suppression in myasthenics, we hypothesize that stimulation of the two mechanisms may have therapeutic potential in MG.

## 1. Introduction

Autoimmune* Myasthenia Gravis* (MG) is the most common T-cell dependent acquired neuromuscular disorder, which in practical terms is characterized by skeletal muscle weakness and fatigability on repetitive use due to autoantibodies directed towards muscle-type nicotinic ACh receptors (nAChR). These antibodies reduce the number of effective receptors to nearly one-third of the normal (reviewed in [1]), leading to a decrease in the safety margin of the neuromuscular transmission, which is particularly relevant during high-frequency nerve activity. By decreasing the generation of postsynaptic action potentials, this condition leads to muscle weakness [2, 3]. Although the therapeutic approach must be individualized according to patients complains, treatment of MG can be divided into symptomatic (acetylcholinesterase inhibitors) and long-term immunosuppression (e.g., corticosteroids, azathioprine, monoclonal antibodies, and removal of the thymus). The overall goal is to restore normal clinical neuromuscular function dealing with the exaggerated immune reaction, while minimizing side effects. Thus, understanding common features that regulate both the safety factor of neuromuscular transmission and the T-cell drive for specific autoantibodies production may be clinically relevant for devising novel and unifying therapeutic strategies to manage MG.

Adenosine (ADO) is an ubiquitous molecule acting as a potent modulator of both neuronal and immunological responses through the activation of adenosine A_2A_ receptors (A_2A_R) [4, 5]. A seminal work from our group at the rat motor endplate demonstrated for the first time that ADO could facilitate the release of neurotransmitters via prejunctional A_2A_R activation, besides the classical neuroinhibitory action mediated by adenosine A_1_ receptors (A_1_R) [4]. Later on, we showed that tonic activation of facilitatory A_2A_R on motor nerve terminals contributes to overcome tetanic depression during high-frequency neuronal firing through the increase in Ca^2+^ influx via Ca_v_1 (L-type) channels [6]. This led us to propose that manipulation of A_2A_R activation could be of clinical interest to preserve neuromuscular transmission in compromised (low safety factor) myasthenic motor endplates. In fact, we proved that impairment of Ca^2+^ influx via Ca_v_1 (L-type) channels due to deficits in A_2A_R tonus contributes to tetanic failure in rats with toxin-induced* Myasthenia gravis* (TIMG) [7]. Moreover, the A_2A_R is now considered an important negative modulator of T-cell function and is also being recognized as a relevant player in the immunopathogenesis of MG [8]. The A_2A_R activation plays a dual role on T cells. This receptor inhibits T-cell receptor (TCR)-mediated signaling, which consequently leads to a decrease in IL-2 production and CD25 expression, and, consequently, to a decline in T-cells proliferation [5, 9]. In addition, activation of the A_2A_R has been shown to increase the expression of FoxP3 in cognate antigen-activated T cells, thus promoting the differentiation of inducible regulatory T cells (T_reg_) [10].

Taking this into consideration, disorders like* Myasthenia gravis* (MG) may benefit from therapeutic strategies targeting common molecular elements involved in both neuromuscular and immunological impairment. Impairment of the A_2A_R neuromodulatory tonus was recently demonstrated in the TIMG rat model [7]. In parallel, Li and collaborators reported decreases in the A_2A_R expression on both CD4^+^ T cells and B cells residing in spleen and lymph nodes following experimental autoimmune* Myasthenia gravis* (EAMG) induction [8]. Thus, one may predict that the adenosinergic A_2A_R-mediated pathway might be a common deficient feature underlying both neuronal and immunological dysfunctions occurring in MG.

AMP dephosphorylation via ecto-5′-nucleotidase/CD73 is the rate limiting step to generate extracellular ADO from released adenine nucleotides. At the rat motor endplate, ADO originating from the catabolism of released adenine nucleotides together with ACh preferentially activates facilitatory A_2A_R on nerve terminals [11–13]. While at the immunological system, formation of ADO by ecto-5′-nucleotidase/CD73 is essential for the immunosuppressant activity of CD4^+^CD25^+^FoxP3^+^  T_reg_ [14]. The pivotal role of ecto-5′-nucleotidase/CD73 in controlling the extracellular ADO levels prompted us to investigate its contribution in the pathogenesis of autoimmune* Myasthenia Gravis* (MG) in order to conceive novel therapeutic strategies to manage this relatively frequent, yet highly incapacitating, disease. In this study, we used a rat model of EAMG that best reproduces the features of human MG, in both clinical and histopathological terms.

## 2. Materials and Methods

### 2.1. Induction and Clinical Assessment of EAMG

Female Wistar rats, weighting approximately 100 g (Charles River, Barcelona, Spain) were kept at a constant temperature (21°C) and a regular light (06.30–19.30 h)—dark (19.30–06.30 h) cycle, with food and water* ad libitum* and randomly divided into three groups (naïve, control, and EAMG). Under general anesthesia, with ketamine (75 mg/kg) and medetomidine (100 mg/kg) by intraperitoneal administration, rats in the EAMG group were immunized by subcutaneous injection at four sites (two hind footpads and shoulders) with 50 *μ*g of R97-116 peptide (DGDFAIVKFTKVLLDYTGHI, JPT Peptide Technologies GmbH), a synthetic peptide corresponding to a specific region on the *α* subunit of the rat nicotinic AChR, made up in complete Freund's adjuvant (CFA) (Sigma, St. Louis, MO, USA). Injections were performed on day 0 and were boosted on day 30 with the same peptide in incomplete Freund's adjuvant (IFA) [15]. The control group was immunized with CFA and IFA emulsions, respectively, containing phosphate-buffered saline (PBS) instead of the nAChR R97-116 peptide at the respective time points. Animals in the naïve group were left untreated. Evaluation of disease manifestations in immunized rats was performed by testing muscular weakness. Clinical scoring was based on the presence of tremor and hunched posture and muscle strength by grip strength test (BIOSEB, France), and fatigability was assessed after exercise (repetitive paw grips on the cage grid). Disease severity was graded as follows: grade 0, normal strength and no fatigability; grade 1, mildly decreased activity and weak grip or cry; grade 2, clinical signs present at rest; grade 3, severe clinical signs at rest, no grip, and moribund; and grade 4, death [15]. Each animal was weighted and evaluated for disease manifestation twice weekly until sacrifice by decapitation on day 42 [15]. Animal handling and experiments were in accordance with the guidelines prepared by Committee on Care and Use of Laboratory Animal Resources (National Research Council, USA) and followed the European Communities Council Directive (86/609/EEC). All the animals included in this study were submitted to the same experimental procedure.

### 2.2. Serum Adenosine Deaminase Activity

The whole blood was collected from the three different groups of rats after decapitation and total serum adenosine deaminase (ADA) activity was determined at 37°C by an enzymatic spectrophotometric method on a Cobas Mira S autoanalyser (Roche Diagnostics, Switzerland), according to the method of Giusti [16].

### 2.3. Muscle Contraction Recordings

The experiments were performed using either left or right phrenic nerve-hemidiaphragm preparations (4–6 mm width). Each muscle was superfused with gassed (95% O_2_-5% CO_2_) Tyrode solution (pH 7.4) containing (mM) NaCl 137, KCl 2.7, CaCl_2_ 1.8, MgCl_2_ 1, NaH_2_PO_4_ 0.4, NaHCO_3_ 11.9, glucose 11.2, and choline 0.001, at 37°C. Tension responses were recorded isometrically at a resting tension of 50 mN with a force transducer and displayed on a Hugo-Sachs (Germany) recorder. Supramaximal intensity rectangular pulses of 40 *μ*s duration and a current strength of 8 mA were applied to phrenic nerve. This was done to achieve synchronization of phrenic motoneuron firing in order to reduce the number of silent units. Pulses were generated by a Grass S48 (USA) stimulator coupled to a stimulus isolation unit (Grass SIU5) operating in a constant current mode. Tetanic failure (fatigue) of hemidiaphragm muscle contractions was achieved using high frequency (50 Hz) intermittent (17 pulses per sec, during 3 minutes) nerve stimulation [17]. The percentage of contractile reduction force was calculated by assessing the percentage of variation of the peak force at the last train (applied at 180 seconds of stimulation) comparatively to the peak force observed at the beginning of the intermittent stimulation.

### 2.4. [^3^H]-ACh Release Experiments

The procedures used for labeling the preparations and measuring evoked [^3^H]-acetylcholine ([^3^H]-ACh) release have been previously described [4, 12]. Briefly, phrenic nerve-hemidiaphragm preparations were mounted in 3 mL capacity Perspex chambers heated to 37°C. Nerve terminals were labeled for 40 min with 1 *μ*M [^3^H]-choline (specific activity 2.5 *μ*Ci/nmol) under electrical stimulation at a frequency of 1 Hz (0.04 ms duration, 8 mA). The phrenic nerve was stimulated with a glass-platinum suction electrode placed near the first division branch of the nerve trunk to avoid direct contact with muscle fibers. Washout of the preparations was performed for 60 min, by superfusion (15 mL/min) with Tyrode's solution supplemented with the choline uptake inhibitor, hemicholinium-3 (10 *μ*M). Release of [^3^H]-ACh was evoked by electrical stimulation of the phrenic nerve with 750 pulses applied at 5 Hz frequency (see stimulation conditions on myographic recordings section). Two stimulation periods were used: at 12 min (*S*_1_) and at 39 min (*S*_2_) after the end of washout (time zero). Test drugs were added 15 min before *S*_2_ and were present up to the end of the experiments. Tritium outflow was evaluated by liquid scintillation spectrometry (% counting efficiency: 40 ± 2%) after appropriate background subtraction using 2 mL bath samples collected automatically every 3 min. After the loading and washout periods, the preparation contained (5542 ± 248) × 10^3^ disintegrations per minute per gram (DPM/g) wet weight of tissue and the resting release was (132 ± 12) × 10^3^ DPM/g (*n* = 8). The fractional release was calculated to be 2.38 ± 0.14% of the radioactivity present in the tissue at the first collected sample. The evoked release of [^3^H]-ACh was calculated by subtracting the basal tritium outflow from the total tritium outflow during the stimulation period [12]. The change in the ratio between the evoked [^3^H]-ACh released during the two stimulation periods (*S*_2_/*S*_1_) relative to that observed in control situations (in the absence of test drugs) was taken as a measure of the effect of the tested drugs.

### 2.5. Kinetics of the Extracellular AMP Catabolism by HPLC (UV Detection)

The extracellular AMP catabolism was evaluated, at 37°C, on phrenic-nerve hemidiaphragm preparations from naïve, control, and EAMG rats. After a 30 min equilibration period, the organ bath was emptied and 2 mL of a 30 *μ*M AMP in gassed Tyrode's solution was added to the preparations at time zero. Samples of 75 *μ*L were collected from the bath at different times up to 45 min for HPLC with UV detection (HPLC-UV, LaChrome Elite, Hitachi, Merck, Germany) analysis of the variation of substrate disappearance and product formation [13, 18]. In all experiments, the concentration of products at the different times of sample collection was corrected by subtracting the concentration of products in samples collected from the same preparation incubated without adding substrate. Only IMP, inosine (INO), and hypoxanthine (HX) were spontaneously released from the preparations in concentrations that did not exceed 1 *μ*M [13]. There was no spontaneous degradation of AMP at 37°C in the absence of the preparation. Concentration of the substrate and products were plotted as a function of time (progress curves). The following parameters were analyzed for each progress curve: half-life time (*t*_1/2_) of the initial substrate, time of appearance of the different concentrations of the products, and concentration of the substrate. At the end of the experiments, the remaining incubation medium was collected and used to quantify the lactate dehydrogenase activity. The negligible activity of this enzyme at the end of the experiments was an indication of the integrity of the cells during experimental period.

### 2.6. Release of Endogenous Adenosine by HPLC (Diode Array Detection)

Experiments were performed using an automated perfusion system for sample collecting for given time periods, therefore improving the efficacy of HPLC (with diode array detection) analysis. After a 30 min equilibration period, the preparations were incubated with 1.5 mL gassed Tyrode's solution, which was automatically changed every 3 min by emptying and refilling the organ bath with the solution in use. The preparations were electrically stimulated once, 15 min after starting sample collection (zero time), using 750 pulses delivered at a 5 Hz frequency. In these experiments, only the sample collected before stimulus application and the two samples collected after stimulation, were retained for analysis. Bath aliquots (50–250 *μ*L) were frozen in liquid nitrogen immediately after collection, stored at −20°C (the enzymes are stable for at least 4 weeks), and analyzed within 1 week of collection by HPLC with diode array detection (Finigan Thermo Fisher Scientific System LC/DAD, equipped with an Accela Pump coupled to an Accela Autosample, a diode array detector, and an Accela PDA running the X-Calibur software chromatography manager). Chromatographic separation was carried out through a Hypersil GOLD C18 column (5 *μ*M, 2.1 mm × 150 mm) equipped with a guard column (5 *μ*m, 2.1 mm × 1 mm) using an elution gradient composed of ammonium acetate (5 mM, with a pH of 6 adjusted with acetic acid) and methanol. During the procedure the flow rate was 200 *μ*L/min and the column temperature was maintained at 20°C. The autosampler was set at 4°C and 50 *μ*L of standard or sample solution was injected, in duplicate, for each HPLC analysis. In order to obtain chromatograms and quantitative analysis with maximal sensibility, the diode array detection wavelength was set at 259 nm for adenosine. Stimulation-evoked release of adenosine was calculated by subtracting the basal release, measured in the sample collected before stimulation, from the total release of adenosine determined after stimulus application.

### 2.7. Immunofluorescence Staining and Confocal Microscopy Observation

One of the major constrains regarding immunolabelling of the mammalian neuromuscular junction is the presence of abundant intramuscular connective tissue, which conceals the synaptic region leading to poor penetration of the labeling antibodies into the tissue. To circumvent this problem, we pretreated muscle fragments with Tyrode's solution continuously gassed with 95% O_2_-5% CO_2_, containing 0.1% collagenase (type I; Sigma Aldrich) for 30 min, in order to increase the access of the antibodies to the neuromuscular junction. Then, the muscle sections were stretched to all directions and pinned onto petri dishes coated with Sylgard. The tissues were, finally, fixed in PLP solution (paraformaldehyde 2%, lysine 0.075 M, sodium phosphate 0.037 M, and sodium periodate 0.01 M) overnight at 4°C and stored in a cryoprotector solution at −20°C. Using a cryostat (Leica CM1950; Leica Microsystems, Nussloch, Germany) kept at −25°C, serial cross-sections of the muscle strips (45 *μ*m) were cut.

After sectioning, tissue fragments were incubated overnight at 4°C with a blocking buffer solution, consisting in foetal bovine serum 10%, bovine serum albumin 1%, and Triton X-100 1% in PBS. Afterwards the samples were incubated with primary antibodies diluted in the incubation buffer (foetal bovine serum 5%, serum albumin 0.5%, and Triton X-100 0.5% in PBS), at 4°C, for 48 h. For immunofluorescent staining of A_2A_R, the rabbit anti-canine A_2A_R polyclonal antibody (1 : 75; A2aR21-A, Alpha Diagnostics International Inc.) and the mouse anti-human A_2A_R monoclonal (1 : 50; 05-717, Clone 7F6-G5-A2, Chemicon) with cross-reactivity with rat A_2A_R, were incubated overnight. After incubation, the sections were washed in PBS supplemented Triton X-100 0.3% (3 cycles of 10 min). Then, species-specific secondary antibodies were applied to tissues samples overnight, at 4°C, in the dark upon which the samples were incubated for 15 min at room temperature with *α*-bungarotoxin (*α*-BTX) (1 : 1500) conjugated with tetramethylrhodamine (TMR-BTX) (Molecular Probes) to provide nAChR detection. Finally, tissue samples were mounted on optical-quality glass slides using the antifading agent VectaShield (VectorLabs) and stored at 4°C. Observations were performed and analyzed with a laser-scanning confocal microscope (Olympus FluoView, FV1000, Tokyo, Japan).

The A_2A_R monoclonal antibody (05-717, Clone 7F6-G5-A2, Chemicon) recognizes an epitope in the third intracellular loop. It detected A_2A_R in paraformaldehyde-fixed mouse brain striatal sections; no signal was observed in A_2A_R knockout mice (see http://www.emdmillipore.com/PT/en/product/Anti-Adenosine-Receptor-A2a-Antibody%2C-clone-7F6-G5-A2,MM_NF-05-717). To test the specificity of the antibody for the A_2A_R (AlphaDiagnostics, A2aR21-A), some sections were processed with the primary antibody preadsorbed with a control antigen corresponding to a 30-amino-acid sequence of the intracellular C-terminus of the canine A2aR/ADORA2A (Gene Accession # P11617) (A2aR21-P, Alpha Diagnostics International Inc., San Antonio, TX, USA, http://www.4adi.com/objects/catalog/product/extras/A2aR21-S-A-P.pdf). Preadsorption was performed by incubating the A_2A_R primary antibody overnight at 4°C with 10-fold molar excess of the antigen peptide sequence. Sections were then processed as described earlier with the preabsorbed antiserum and with the normal antiserum, in parallel. During documentation of A_2A_R preabsorption controls, settings on the confocal microscope were adjusted appropriately to show A_2A_R-immunoreactivity for sections that were processed normally (no preabsorption) and these settings were maintained when documenting pre-absorption controls to minimize bias, during capture and printing of digital images.

### 2.8. CD4^+^ T Cell Enrichment

Popliteal and inguinal lymph nodes were removed from naïve, control, and EAMG animals and homogenized to single-cell suspensions. CD4^+^ T lymphocytes-enriched suspensions were prepared by incubation of total lymph node cells with anti-CD4 magnetic microbeads (Miltenyi Biotech) that were further separated on LS columns (Miltenyi Biotech) according to the manufacturer's recommendations. The proportions of CD4^+^ cells in the enriched suspensions typically ranged from 80 to 90%.

### 2.9. T-Cells Analysis by Flow Cytometry

Different combinations of antibodies were used to characterize cells derived from the different groups of animals. 1 × 10^6^ cells/mL of CD4^+^ T cell-enriched suspensions were blocked with 10% (V/V) mouse and rabbit sera before incubation with the following primary antibodies: FITC-conjugated anti-rat CD4 (1 : 100, eBioscience, clone OX35), PE-conjugated anti-rat CD25 (1 : 100; eBioscience, clone OX39), and rabbit anti-rat CD73 antibody (1 : 750; kindly provided by Professor Jean Sévigny, Univ. Laval, Québec, QC, Canada; can be obtained at http://www.ectonucleotidases-ab.com) during 30 min at 4°C in the dark. The anti-CD73 antibody was revealed with biotin-conjugated anti-rabbit IgG (Fc specific) (1 : 750, Sigma Aldrich) followed by incubation with Streptavidin PE-Cy7 (1 : 100; eBioscience). The specificity of the anti-rat CD73 antibody was confirmed by immunoblotting, flow cytometry, and immunohistochemistry [19].

For analysis of FoxP3 expression, cells were fixated and permeabilized using the fixation/permeabilization FoxP3 Kit (eBioscience) and labeled with PE-Cy5-conjugated anti-mouse/rat FoxP3 (1 : 100, eBioscience, clone FJK-16s). Isotype matched fluorochrome conjugated mAbs of irrelevant specificity were used as negative controls. The samples were analyzed in an EPICS XL flow cytometer using the EXPO32ADC software (Beckman Coulter, Miami, FL). The collected data files (100 000 events per sample) were converted for analysis, with the CELLQUEST software, v3.2.1f1 by using FACS CONVERT, v1.0 (both from Becton Dickinson, San Jose, CA).

### 2.10. Proliferation Assays

CD4^+^ T cells were isolated from the popliteal and inguinal lymph nodes of control and EAMG animals by using magnetic cell sorting rat CD4^+^ microbeads (Miltenyi Biotech, Inc., Auburn, CA, USA) following the manufacturer's instructions. The CellTrace CFSE Cell Proliferation Kit (Molecular Probes, Invitrogen, Eugene, OR, USA) was used for cell labelling. A CFSE (5-(and-6)-carboxyfluorescein diacetate succinimidyl ester) stock solution (10 mM in DMSO) stored at −20°C was thawed and diluted in PBS with 0.1% BSA to a final concentration of 10 *μ*M. CD4^+^ T cells were resuspended at 2 × 10^6^/mL in PBS with 0.1% BSA and further incubated with an equal volume of the diluted CFSE solution, for 7 min at 37°C. Cells were washed three times with complete RPMI medium. CD4^+^ T cells were plated at 5 × 10^4^/well in U-shape 96-well plates without stimulus or stimulated with 1 *μ*g/mL plate-bound anti-CD3 mAb (clone G4.18) and 1 *μ*g/mL soluble anti-CD28 mAb (clone JJ319) (both from eBioscience). Additionally, cells were supplemented with 30 *μ*M or 100 *μ*M AMP every 12 h, in the absence or presence of either ADA (0.5 U/mL) or ZM241385 (50 nM). Unlabelled stimulated cells were used to define cell autofluorescence. Each condition was set in triplicate and cultures were maintained for 72 h at 37°C and 5% CO_2_. Proliferation was determined based on CFSE fluorescence by flow cytometry analysis. Representative CFSE histograms are presented. The percentage of effect of AMP on CD4^+^ T cells proliferation was calculated by using the mean proliferation index of CD4^+^ T cells in the absence of the drug compared with the proliferation index of CD4^+^ T cells incubated with AMP. When using the modifiers ADA (0.5 U/mL) or ZM 241285 (50 nM), the effect percentage of AMP on CD4^+^ T cells proliferation was calculated by using the mean proliferation index of CD4^+^ T cells in the presence of ADA (0,5 U/mL) or ZM 241285 (50 nM) compared with the proliferation index of CD4^+^ T cells incubated with AMP alone.

### 2.11. Drugs and Solutions

Adenosine deaminase (ADA, type VI, 1803 U/mL, EC 3.3.3.4), adenosine monophosphate (AMP), choline chloride, hemicholinium-3, CFA, and IFA was from Sigma (Sigma, St. Louis, MO, USA), 4-(-2-[7-amino-2-{2-furyl}{1,2,4}triazolo{2,3-a}{1,3,5}triazin-5-yl-amino]ethyl)phenol (ZM 241385) was from Tocris Bioscience (Tocris Bioscience, Bristol, UK). [Methyl-^3^H]choline chloride (ethanol solution, 80 Ci mmol^−1^) was obtained from Amersham International (Amersham, UK). ZM 241385 was made up in dimethyl sulphoxide (DMSO). All stock solutions were stored as frozen aliquots at −20°C. Dilutions of these stock solutions were made daily and appropriate solvent controls were done. No statistically significant differences between control experiments, made in the absence or in the presence of the solvents at the maximal concentrations used, were observed.

### 2.12. Statistics

Results are expressed as mean ± SEM, with *n* indicating the number of animals used for a particular set of experiments. Statistical analysis of data was carried out using paired or unpaired Student's *t*-test or one-way analysis of variance (ANOVA) followed by Dunnett's or Bonferroni's post hoc test. Values of *P* < 0.05 were considered to represent significant differences.

## 3. Results and Discussion

### 3.1. Neurophysiological and Immunological Features of EAMG in the Wistar Rat

Most commonly, EAMG as a model of MG in humans can be induced in rats by immunization with nAChR purified from Torpedo electric organ in CFA and by passive transfer of serum from EAMG rats or from patients with MG [20]. Both in rat EAMG and in human MG, the production of autoantibodies to AChR is dependent on T-cell help [1]. In this study, we used a synthetic peptide corresponding to the R97-116 sequence found in *α* subunit of the rat nAChR in CFA to induce EAMG in Wistar rats [15]. These animals were screened for markers of neuromuscular and immunological imbalance. Rat EAMG is characterized clinically by two distinct phases: a transient acute phase with weakness (affecting predominantly the forelimbs, head, neck, laryngeal, and respiratory muscles) that begins approximately 7 days post immunization (p.i.) and with recovery after 3-4 days and a progressive chronic phase with onset approximately 28 days p.i., where weakness is progressive, often ending in death.

**Figure 1:**
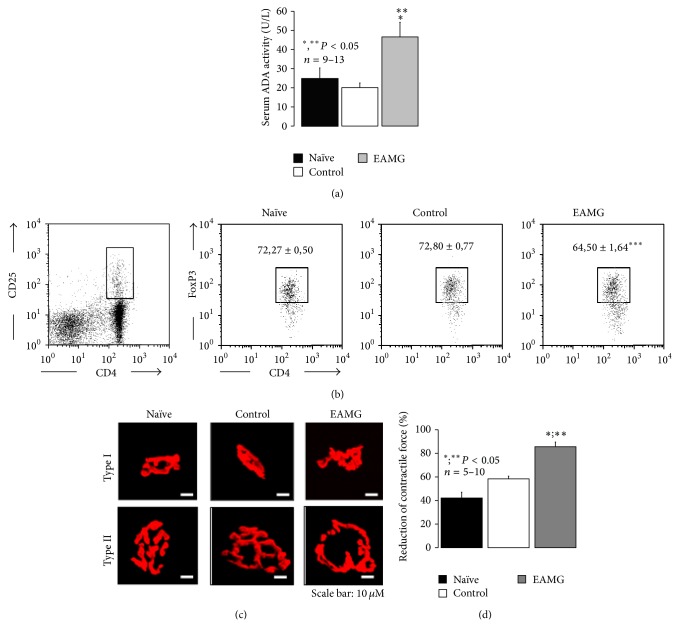
Neuromuscular and immunological deficits of rats with EAMG. Experiments were performed six weeks after immunization with the peptide R97-116 corresponding to the *α*-subunit of nAChR in CFA (EAMG), as compared to age-matched naïve and control littermates. (a) Measurement of serum adenosine deaminase (ADA) activity. Results are mean ± SEM of 9 naïve, 13 control, and 12 EAMG rats. ^*^, ^**^ *P* < 0.05 (one-way ANOVA following Dunnett's modified *t*-test) compared to naïve and control animals, respectively. (b) Flow cytometry analysis of intracellular FoxP3 expression in CD4^+^CD25^+^  T cells collected from popliteal and inguinal lymph nodes from naïve, control, and EAMG rats. Gating strategy to delimit CD4^+^CD25^+^ cells is shown on the left dot plot. Dot plots on the right show FoxP3 expression within gated CD4^+^CD25^+^ cells. Gate inside these dot plots correspond to FoxP3^+^ cells. Dot plots are a representative example of each indicated group. Numbers inside dot plots correspond to mean percentage ± SEM of FoxP3^+^ cells within CD4^+^CD25^+^ population. Statistically significant difference between EAMG (*n* = 13) and both naïve (*n* = 9) and control (*n* = 13) groups is indicated (^***^ *P* < 0.001; one-way ANOVA and Bonferroni's post hoc test). (c) Confocal microscopy micrographs showing type I and type II motor endplates from hemidiaphragm sections labeled with TMR-*α*-BTX* (red)* from naïve, control, and EAMG animals. Scale bar: 10 *μ*m. (d) Reduction of contractile strength of isolated hemidiaphragm preparations from naïve, control, and EAMG animals during a 3 min period of intermittent phrenic nerve stimulation (17 pulses per second delivered at 50 Hz frequency). Results are mean ± SEM of 10 naïve, 6 control, and 5 EAMG rats. ^*^, ^**^ *P* < 0.05 (one-way ANOVA following Dunnett's modified *t*-test) compared to naïve and control animals, respectively.

Previous studies have demonstrated a positive correlation between increase in serum adenosine deaminase (ADA) activity, the enzyme that inactivates ADO into INO, and the clinical score of myasthenic patients [21]. Moreover, symptoms and T cell mediated reactivity have been inversely related to the percentage of CD4^+^CD25^+^FoxP3^+^  T_reg_ cell population in human MG [22]. In this context, we thought it will be interesting to evaluate the total serum ADA activity and the relative proportion of CD4^+^CD25^+^FoxP3^+^  T_reg_ in the cell suspensions obtained from draining popliteal and inguinal lymph nodes in the rat model of EAMG to see if they compare to the human disease.  Figure 1(a) shows that serum ADA activity was significantly (*P* < 0.05) higher (47 ± 8 U/L, *n* = 12) in EAMG animals as compared to both control (20 ± 2 U/L, *n* = 13) and naïve (25 ± 6 U/L, *n* = 9) littermates. The increase in serum ADA activity that we show here for the first time has been considered a hallmark of MG pathophysiology [21], since ADA influences proliferation and differentiation of lymphocytes, especially of T cells [23]. This effect seems to be specific, as the exacerbation of total serum ADA activity was observed only in EAMG, but not in control and naïve animals. In addition, EAMG animals exhibit a significant (*P* < 0.001) reduction in the relative proportion of CD4^+^CD25^+^FoxP3^+^  T_reg_ cells among the CD4^+^CD25^+^  T cell population obtained by draining popliteal and inguinal lymph nodes (64.50 ± 1.64%, *n* = 13) as compared to both control (72.80 ± 0.77%, *n* = 13) and naïve (72.27 ± 0.50%, *n* = 9) animals (Figure 1(b)). These findings obtained by immunizing Wistar rats with the R97-116 peptide sequence of the nAChR *α* subunit are in agreement with those verified in the EAMG model induced in Lewis rats either with the Torpedo nAChR [24] or with the same rat R97-116 peptide [25], as well as in the human MG [22, 26]. No significant differences (*P* > 0.05) were found among the three animal groups in the percentage of total CD25^+^ cells within the CD4^+^ T cell population** (**7.29 ± 0.40%, 8.17 ± 0.28% and 8.56 ± 0.46% for naïve, control, and EAMG groups, resp.). Interestingly, long term administration of enzyme replacement therapy with pegylated bovine ADA (PEG-ADA) has been associated to manifestations of immune dysregulation including autoimmunity [27]. The increased turnover of ADO by ADA seems to interfere with the CD4^+^CD25^+^FoxP3^+^  T_reg_ mediated control of immune responses since T_reg_ cells isolated from PEG-ADA-treated patients are reduced in number and show decreased activity [27].

Neuromuscular transmission failure in autoimmune MG results from an antibody attack to postsynaptic muscle nAChRs decreasing their number and causing a disorganization of receptor clusters at the motor endplate (reviewed in [1]). Therefore, we decided to evaluate the occurrence of similar morphological changes at diaphragm motor endplates of EAMG rats by immunofluorescence confocal microscopy. Previous studies from our and many other laboratories demonstrated that immunofluorescence labeling of postsynaptic *α*1-subunits of nAChR with *α*-bungarotoxin conjugated with tetramethylrhodamine was instrumental to evaluate histological modifications of the neuromuscular junction from myasthenic animals (see e.g., [7]). The diaphragm was chosen because it is a highly active skeletal muscle (duty cycle ~25–40%) of mixed fiber composition.  Figure 1(c) shows typical motor endplates of slow (type I) and fast (type II) muscle fibers from naïve, control, and EAMG rats labeled with tetramethylrhodamine conjugated with *α*-bungarotoxin. Planar (two-dimensional) area measurements showed expected size differences. Those at type I diaphragm muscle fibers were smaller and with less exuberant postsynaptic folding compared to type II fibers. Motor endplates of EAMG animals exhibit significant morphological alterations as compared to naïve and control rats. These changes were similar to the ones observed in samples from MG patients [28]. Changes, which include significant (*P* < 0.05) reductions in the total area of nAChR labeling per endplate, were observed predominantly on type II fibers (Figure 1(c)). Data are in agreement with previous findings showing a reduction in the number of effective nAChR receptors. Most probably, autoantibodies present in EAMG animals bind to the nAChR to cause receptor internalization and degradation. The antibody-nAChR complex also binds to complement resulting in damage of the postsynaptic membrane, which typically has fewer secondary synaptic folds and a widened synaptic cleft that leads to loss of functional receptors (Figure 1(c); see e.g., [29]).

These morphological changes reduce the safety margin of neuromuscular transmission. Considering that the reduced skeletal muscle strength during repetitive nerve stimulation reflects the neuromuscular/immunological imbalance operating in EAMG, we performed myographic recordings using diaphragm preparations stimulated indirectly via the phrenic nerve trunk under fatigue conditions. These were produced by high-frequency (50 Hz) intermittent (17 pulses per sec, during 3 minutes) phrenic nerve stimulation [17].  Figure 1(d) shows that muscle fatigue was significantly (*P* < 0.05) more intense in EAMG animals than in both naïve and control littermates.

Overall these data suggest that immunization of rats with a single peptide fragment homologous to a region of the *α*-subunit of the nAChR leads to pathophysiological and clinical features observed in human MG. Thus, we are confident that the EAMG rat model might be extensively used to unravel the pathogenesis of MG and to explore novel therapeutic strategies to manage this disease [30].

### 3.2. Altered Expression of Ecto-5′-nucleotidase/CD73 in CD4^+^ T Cell Subsets from Lymph Nodes of EAMG Rats

**Figure 2:**
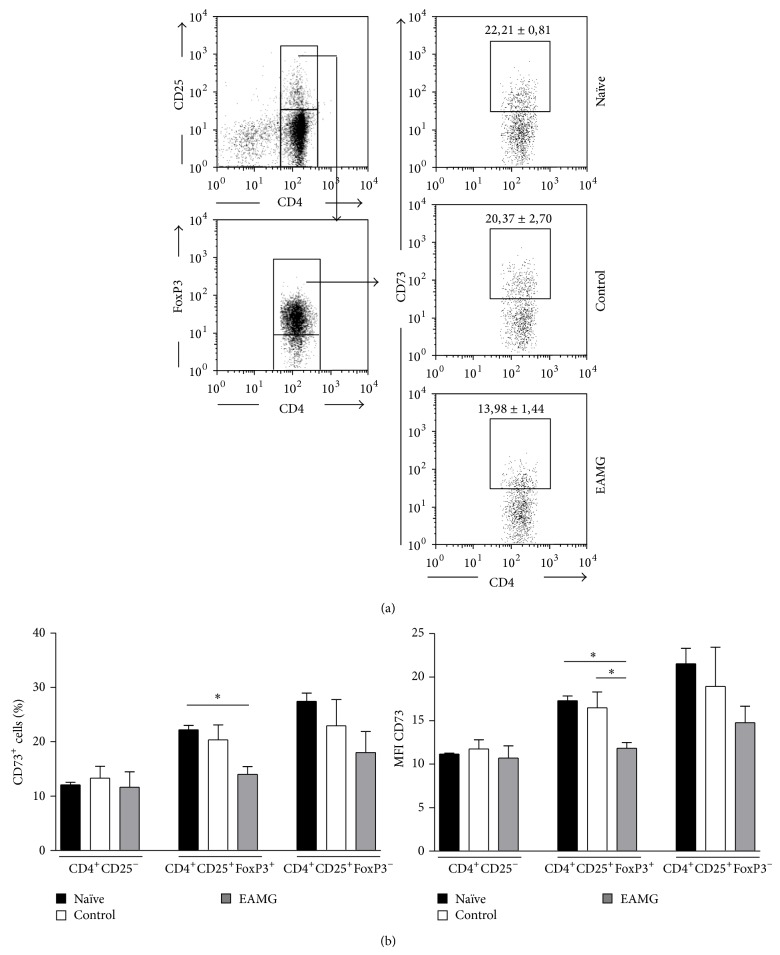
(a) Flow cytometry analysis of surface CD73 expression on CD4^+^CD25^+^FoxP3^+^  T cells of popliteal and inguinal lymph nodes from naïve, control, and EAMG animals. Gating strategies are indicated on dot plots on the left. Dot plots on the right show CD73 expression within CD4^+^CD25^+^FoxP3^+^  T cells and are a representative example of each indicated group. Numbers inside dot plots correspond to mean percentage ± SEM of CD73^+^ cells. (b) Percentage of CD73^+^ cells (left) and mean fluorescence intensity (MFI) due to CD73 (right) staining within the indicated cell populations. Bars represent means ± SEM of 5 experiments for each animal group. ^*^ *P* < 0.05, ^**^ *P* < 0.01 (one-way ANOVA and Bonferroni's post hoc test) compared to naïve and control animals, respectively.

Adenosine formation from released adenine nucleotides via ecto-5′-nucleotidase/CD73 expressed on T_reg_ cells regulates the function of stimulated T cells through A_2A_R activation [14, 31]. In a recent study, Li et al. [8] reported a reduction in the expression of A_2A_R on both T and B cells residing in lymph nodes of EAMG animals. Here, we focus our attention on the distribution of ecto-5′-nucleotidase/CD73 in CD25^−^, CD25^+^Foxp3^+^ and CD25^+^Foxp3^−^ T cells among the total CD4^+^ T cells population obtained from inguinal and popliteal lymph nodes of EAMG animals as compared to their naïve and control littermates.  Figure 2 shows a significant (*P* < 0.05) reduction in the proportion of CD4^+^CD25^+^FoxP3^+^  T_reg_ cells expressing CD73 in popliteal and inguinal lymph nodes of EAMG animals (13.98 ± 1.44, *n* = 5) as compared to naïve (22.21 ± 0.81, *n* = 5) and control (20.23 ± 2.70, *n* = 5) groups. A decrease was also observed in the mean fluorescence intensity (MFI) due to CD73 staining on CD4^+^CD25^+^FoxP3^+^  T_reg_ cells of the EAMG group (11.84 ± 0.63) as compared to the other assessed groups, both naïve (17.31 ± 0.51) and control (13,29 ± 1.47) (Figure 2). In contrast, no significant differences were found in the MFI and in the proportion of CD73-expressing effector (CD4^+^CD25^+^FoxP3^−^) T cells and nonactivated (CD4^+^CD25^−^) T cells, among all groups analyzed.

The decrease in the proportion of cells expressing ecto-5′-nucleotidase/CD73, within the CD4^+^CD25^+^FoxP3^+^  T cell population isolated from the lymph nodes of myasthenic rats, might have functional repercussions given that the ecto-5′-nucleotidase/CD73 pathway is responsible for increasing the production of ADO by T_reg_ cells, which exerts an immunosuppressive action on activated T cells via A_2A_R activation [14, 31]. The reduction in ecto-5′-nucleotidase/CD73 expression in T_reg_ cells from EAMG rats suggests that the regulatory loop of ADO accumulation in close proximity of these cells might be impaired. This was hypothesized because deficits in tonic A_2A_R activation of T_reg_ have been shown to decrease FoxP3 mRNA production [10] leading to insufficient expression of ecto-5′-nucleotidase/CD73 [32]. Indeed, Nessi et al. [33] put forward the hypothesis that the inability of CD4^+^CD25^+^FoxP3^+^  T_reg_ to revert ongoing EAMG may be due to inadequate control of activated T cells leading to B-cell activation and differentiation into nAChR antibody-secreting plasma cells. In agreement with this hypothesis, we report here that incubation of CD4^+^ T cells with ZM241385 (50 nM, a selective A_2A_R antagonist) or with ADA (0.5 U/mL, the enzyme that inactivates ADO into inosine) significantly (*P* < 0.05) enhanced cells proliferation, respectively, by 21 ± 3% (*n* = 4) and 18 ± 4% (*n* = 4) in control animals, but removal of the A_2A_R tonus by endogenous ADO failed to cause similar effects in cells from EAMG rats (data not shown). Moreover, T_reg_ cells inability to sustain relatively high concentration of ADO can be further aggravated by increased serum ADA activity [27].

**Figure 3:**
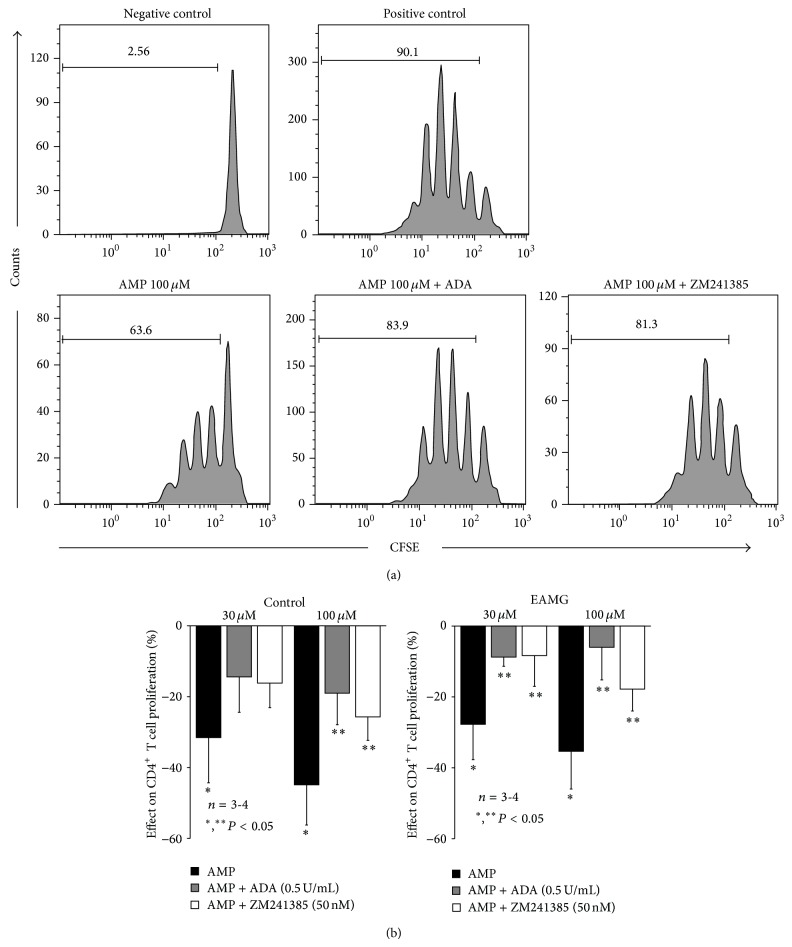
(a) Flow cytometric evaluation of plated anti-CD3 and soluble anti-CD28 mAbs (1 *μ*g/mL) induced proliferative response of 5 × 10^4^ CFSE-labelled CD4^+^T cells, sorted from popliteal and inguinal lymph nodes of a control animal, cultured for 3 days in the absence (positive control) or presence of AMP (100 *μ*M), AMP (100 *μ*M) plus ADA (0,5 U/mL), or AMP (100 *μ*M) plus ZM241385 (50 nM), as indicated. Negative control corresponds to unstimulated cells (no mAbs added). Numbers within histograms correspond to the percentage of cells that divided at least once. Results shown are a representative example of 3 to 4 independent experiments performed in different animals. (b) AMP induced inhibition of CD4^+^ T cell proliferation obtained from popliteal and inguinal lymph nodes from control and EAMG animals. Bars represent means ± SEM of 3 control and 4 EAMG animals. ^*^ *P* < 0.05 compared to the absence of AMP and ^**^ *P* < 0.05 compared to the AMP effect (one-way ANOVA and Bonferroni's post hoc test).

The recovery of extracellular ADO levels may be an attractive pharmacological therapy to restore the immunological competence in myasthenic animals. Data shown in Figure 3 demonstrate that A_2A_R-mediated suppression of CD4^+^ T cells proliferation can be rehabilitated almost to control levels when cells isolated from EAMG rats were supplemented with the nucleoside precursor, AMP (30–100 *μ*M). That is, AMP (30–100 *μ*M) concentration dependently suppressed proliferation of CD4^+^ T cells isolated from both control and EAMG animals. The immune suppressive effect of AMP (30–100 *μ*M) was significantly (*P* < 0.05) attenuated when it was applied together with ZM241385 (50 nM) or ADA (0.5 U/mL) (Figure 3), suggesting that the nucleotide has to be hydrolyzed into ADO, which acts via A_2A_R to suppress CD4^+^ T-cells proliferation. Results show that activation of A_2A_R rehabilitated by AMP dephosphorylation into ADO, via ecto-5′-nucleotidase/CD73, can restore immune competence in EAMG rats, strengthening the hypothesis that immune suppression deficits reside on low ADO generation from released adenine nucleotides to levels below those required to tonically activate A_2A_R.

### 3.3. Recovery of the Facilitatory A_2A_R-Mediated Tonus by ADO Generated via Ecto-5′-nucleotidase/CD73 at the Motor Endplate of EAMG Rat

**Figure 4:**
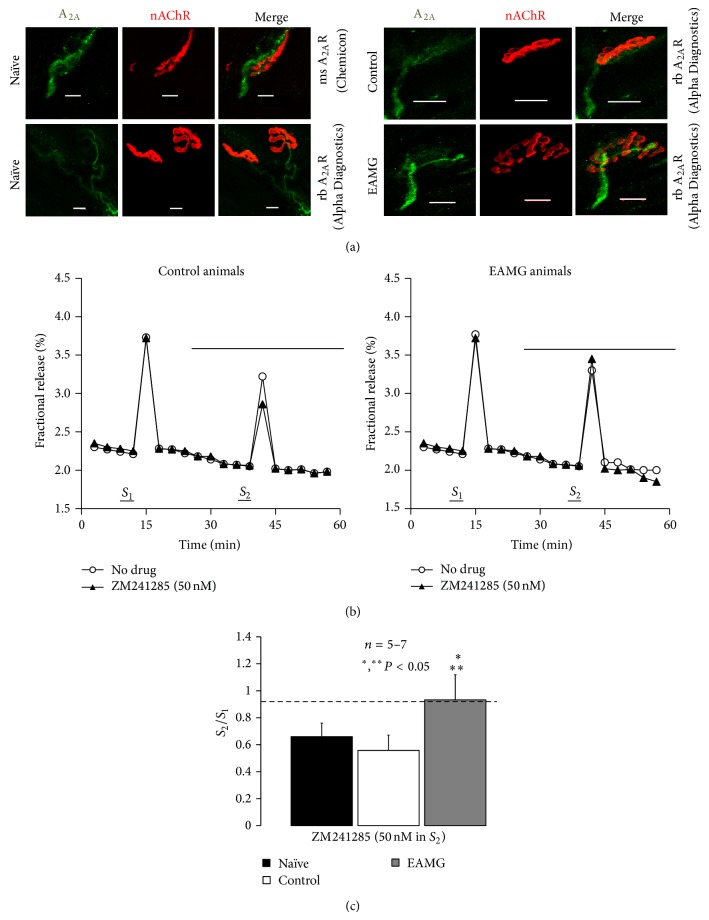
Tonic activation of A_2A_R is significantly impaired at motor endplates of myasthenic rats. (a) Confocal micrographs showing immunoreactivity against A_2A_R (*green*) on motor endplates from rat hemidiaphragms labeled with TMR-*α*-BTX (*red*) from naïve, control, and EAMG rats. Scale bar: 10 *μ*m. Two distinct A_2A_R antibodies, AlphaDiagnostics (A2aR21-P) and Chemicon (05-717, Clone 7F6-G5-A2), were used was indicated. (b) Time course of tritium outflow from phrenic nerve terminals from control and EAMG animals taken from typical experiments in the absence (no drug, open circles) and in the presence of the selective A_2A_R antagonist, ZM241285 (50 nM) (filled triangles). [^3^H]-ACh release was elicited by stimulating the phrenic nerve trunk with 750 pulses delivered with a frequency of 5 Hz at the indicated times (*S*_1_ and *S*_2_). ZM241285 (50 nM) was applied 15 min before *S*_2_. (c) Modification of the *S*_2_/*S*_1_ ratio caused by ZM241285 (50 nM) in naïve, control, and EAMG rats. Each column represents pooled data from five (naïve and control) and seven (EAMG) animals. The vertical bars represent mean ± SEM. ^*^, ^**^ *P* < 0.05 (one-way ANOVA followed by Dunnett's modified *t*-test) when compared to naïve and control (CFA) rats.

The presence of A_2A_R at the motor endplate of naive rats was demonstrated by immunofluorescence confocal microscopy using two distinct commercially available antibodies (Figure 4, for details see Materials and Methods). The antibody from Alpha Diagnostics International Inc. (A2aR21-A) is directed against a 30-amino-acid peptide (A2aR21-P) in the intracellular C-terminus of the canine A_2A_R, which is only 43% conserved in the rat A_2A_R (see http://www.4adi.com/objects/catalog/product/extras/A2aR21-S-A-P.pdf). Although it has been shown to cross-react with the rat A_2A_R, we are aware that specificity has to be proven in transfected cells or in knock-out animals. For this reason, we used an alternative antibody from Chemicon (05-717), which was designed to recognize an epitope in the third intracellular loop of the human recombinant A_2A_R (Clone 7F6-G5-A2) while cross-reacting significantly with the rat receptor (see e.g., [34]); immunostaining with this antibody was abrogated in the striatum of the A_2A_R knock-out mouse (see http://www.emdmillipore.com/PT/en/product/Anti-Adenosine-Receptor-A2a-Antibody%2C-clone-7F6-G5-A2,MM_NF-05-717). Data show that the immunostaining pattern, with both antibodies, A2aR21-A and 05-717, was quite similar. Preadsorption with the peptide A2aR21-P abrogated staining with the A2aR21-A antibody while keeping the same acquisition settings on the confocal microscope. Taking this into account and the requirements for colocalization tests conducted in parallel, whose results are beyond the scope of this study, we continued the experiments with the antibody from Alpha Diagnostics International Inc. (A2aR21-A).

Figure 4(a) shows that immunoreactivity against A_2A_R is located predominantly on nerve axons and presynaptic buttons in close opposition to the motor endplate region, which is highly enriched in nAChRs labeled with *α*-bungarotoxin conjugated with tetramethylrhodamine. We found no evidence to suggest localization of A_2A_R in skeletal muscle fibers. No clear distinction was observed on the A_2A_R immunoreactivity pattern between the three animal groups, naïve, control, and EAMG (Figure 4(a)). Despite this, discrete changes in the expression of A_2A_R in EAMG rats cannot be ruled out and deserve further attention.

Using a neurochemical approach, we evaluated the A_2A_R-mediated tonus on [^3^H]-ACh release from hemidiaphragm preparations indirectly stimulated via the phrenic nerve trunk with 750 supramaximal repetitive pulses delivered with frequency of 5 Hz (Figures 4(b) and 4(c)). Selective blockade of A_2A_R with ZM241385 (50 nM) decreased nerve-evoked [^3^H]-ACh release roughly by 30% in both naïve and control rats (Figures 4(b) and 4(c)), indicating that endogenous ADO exerts a predominant facilitatory tonus, via the activation of A_2A_R, on neuromuscular transmission (see e.g., [4, 12]). Conversely, ZM241385 (50 nM) failed to decrease the evoked [^3^H]-ACh release in EAMG animals (Figure 4(c)). Data from confocal microscopy and neurochemical studies suggest that despite the fact that A_2A_Rs are present on motor nerve terminals of EAMG animals (Figure 4(a)), activation of these receptors by endogenously generated ADO is significantly impaired (Figures 4(b) and 4(c)).

**Figure 5:**
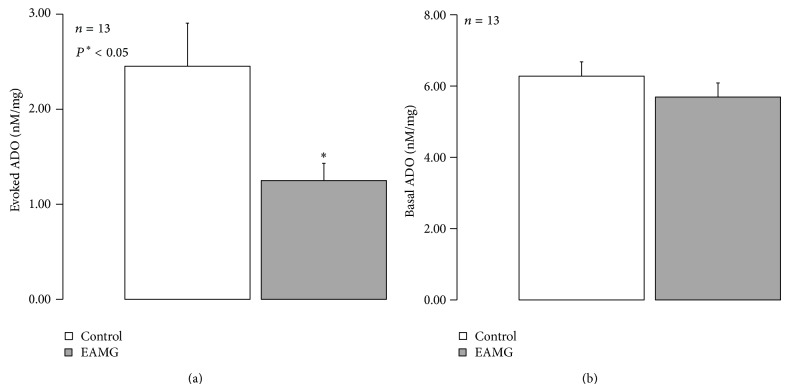
The amount ADO released upon phrenic nerve stimulation is lower in EAMG animals. The ordinates represent the (a) evoked release of ADO upon phrenic nerve trunk electrical stimulation (750 pulses applied at 5 Hz frequency) and (b) basal ADO quantified by HPLC(diode array detection). Nerve-evoked release of ADO was calculated by subtracting the basal release, measured in the sample collected before stimulation, from the total release of adenosine determined after stimulus application. The data are means ± S.E.M. of 13 animals of each group (naïve, control, and EAMG).

Previous studies from our group demonstrated that amplification of transmitter release caused by A_2A_R becomes evident at high levels of synaptic ADO accumulation [12, 35]. Therefore, one may hypothesize that deficits in ADO accumulation at the synaptic cleft may contribute to the loss of ADO neurofacilitation in myasthenic animals.  Figure 5(a) shows that EAMG animals accumulate smaller amounts of ADO (1.25 ± 0.18 nM/mg of tissue, *n* = 13) following phrenic nerve stimulation compared to control animals (2.45 ± 0.45 nM/mg of tissue, *n* = 13). No changes were observed between groups regarding the baseline levels of ADO (Figure 5(b)).

**Figure 6:**
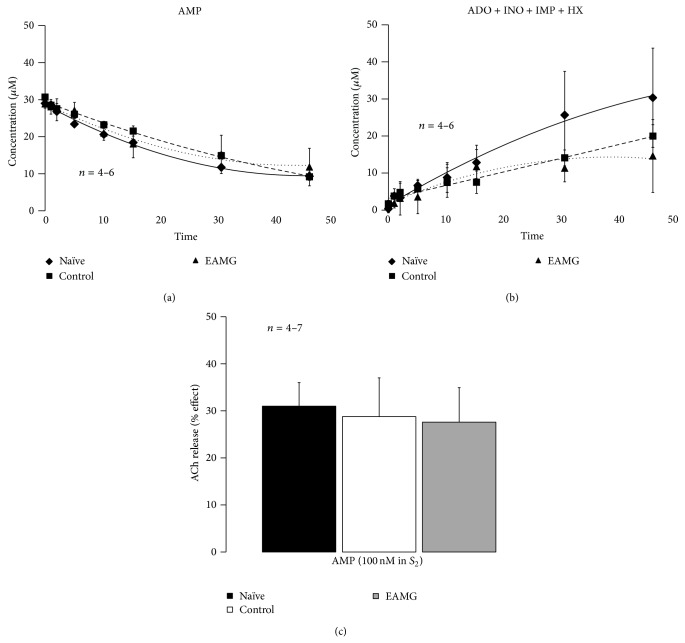
Dephosphorylation of AMP, to ADO via ecto-5′-nucleotidase/CD73, facilitates the release of [^3^H]-ACh from stimulated phrenic motor nerve terminals of naïve, control, and EAMG rats. (a) and (b) show the kinetics of the extracellular AMP (30 *μ*M) catabolism (a) and formation of ADO plus nucleoside derivatives (inosine (INO) and hypoxanthine (HX)) (b) in hemidiaphragm preparations from naïve, control, and EAMG rats. AMP (30 *μ*M) was added to the preparation at zero time; samples were collected from the bath at indicated times on the abscissa and retained for HPLC analysis. Data shown are averages pooled from 4 naïve, 6 control, and 6 EAMG animals. The vertical bars represent SEM and are shown when they exceed the symbols in size. (c) Facilitatory effects of AMP (100 *μ*M) on evoked [^3^H]-ACh release (5 Hz, 750 pulses, *S*_1_ and *S*_2_) from hemidiaphragm preparations from naïve, control, and EAMG rats. AMP (100 *μ*M) was applied 15 min before *S*_2_. Each column represents pooled data from 4 (naïve and control) and 7 (EAMG) animals. The vertical bars represent mean ± SEM.

Considering that ADO originating from the catabolism of released adenine nucleotides together with ACh preferentially activates facilitatory A_2A_R on nerve terminals [11, 12] and knowing the regulatory potential of ecto-5′-nucleotidase/CD73 for adenosine formation, we thought it was important to compare the activity of this enzyme at the skeletal neuromuscular junction of naïve, control, and EAMG animals by assessing the time course of the extracellular catabolism of AMP (30 *μ*M) (Figure 6(a)). The kinetics of the extracellular catabolism of AMP (30 *μ*M) and ADO formation in EAMG animals was very similar to that observed in control rats (Figures 6(a) and 6(b), resp.). No significant changes (*P* > 0.05) were detected when comparing the half degradation time of extracellular AMP (30 *μ*M) in EAMG (22 ± 3 min, *n* = 6), control (26 ± 1 min, *n* = 6), and naïve (26 ± 3 min, *n* = 4) rats (cf. [13]).

Given the similarity of extracellular adenosine formation via ecto-5′-nucleotidase/CD73 among the three animal groups, we tested whether AMP could restore the ADO tonus required to sustain transmitter release from stimulated motor nerve terminals of EAMG rats, as we observed in the toxicological MG rat model [7].  Figure 6(c) shows that exogenously added AMP (100 *μ*M) consistently enhanced nerve-evoked [^3^H]-ACh release from hemidiaphragm preparations of EAMG animals (28 ± 7%, *n* = 7) by a similar amount to that obtained in naïve (31 ± 5%, *n* = 4) and control (29 ± 8%, *n* = 4) muscles. Pretreatment with ADA (0.5 U/mL), the enzyme, that inactivates ADO into INO, prevented the facilitatory effect of AMP (100 *μ*M) on both control (−4 ± 6%, *n* = 4) and EAMG (10 ± 9%, *n* = 4) animals. Likewise, selective blockade of A_2A_R with ZM241285 (50 nM) also attenuated AMP-induced facilitation of evoked [^3^H]-ACh release in control (9 ± 5%, *n* = 4) and EAMG (15 ± 3%, *n* = 4) animals (data not shown). These results indicate that the facilitatory effect of AMP (100 *μ*M) on evoked transmitter release requires its conversion, into ADO and, subsequent, activation of A_2A_R.

## 4. Conclusion

**Figure 7:**
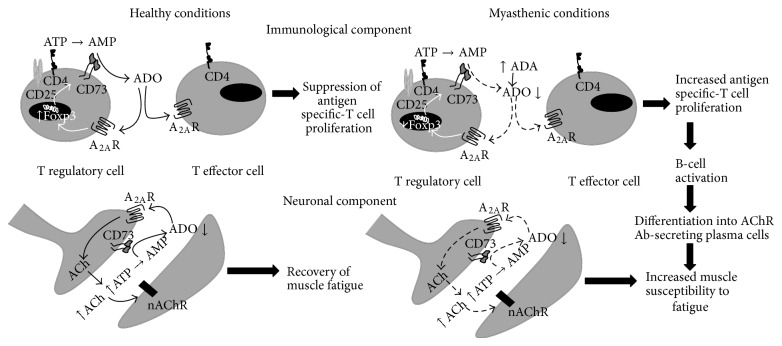
Participation of the adenosinergic system on neuroimmunological deficits present in EAMG rats. In healthy animals, A_2A_R activation by ADO generated from the catabolism of released nucleotides via ecto-5′-nucleotidase/CD73 downmodulates T effector (CD4^+^CD25^+^FoxP3^−^) cells proliferation in response to specific antigens by increasing the activity of T_reg_ (CD4^+^CD25^+^FoxP3^+^) cells expressing FoxP3-dependent gene products, like ecto-5′-nucleotidase/CD73. At the neuromuscular junction, ecto-5′-nucleotidase/CD73 activity leads to the formation of ADO from released ATP (from both nerve and muscle), which facilitates acetylcholine release via prejunctional A_2A_R activation that is necessary to resist tetanic depression. In EAMG rats, increases in serum adenosine deaminase (ADA) together with ecto-5′-nucleotidase/CD73 in T_reg_ (CD4^+^CD25^+^FoxP3^+^) cells lead to insufficient amounts of extracellular ADO. The lack of the A_2A_R immunosuppressive tonus contributes to the loss of peripheral tolerance to nAChR. Thus, increases in the proliferation of antigen-specific T effector cells triggers B cells differentiation into plasma cells and secretion of antibodies directed towards motor endplates nAChR clusters. This antibody attack leads to nAChR internalization/degradation and to complement-mediated morphological changes of the myasthenic postsynaptic membrane (e.g., fewer secondary synaptic folds, widening of the synaptic cleft). These changes contribute to neuromuscular transmission failure, which is further aggravated by deficits in the production of extracellular ADO, probably from released adenine nucleotides, namely, ATP. Impairment of tonic A_2A_R-mediated facilitation of transmitter Impairment of tonic A_2A_R-mediated facilitation of transmitter release turns myasthenic skeletal muscles unable to resist fatigue.

ADO is an extracellular signaling nucleoside that has a unique dynamic role in the regulation of synaptic neurotransmission [4, 6] and immunosuppressive [5, 9, 10] responses, via the activation of A_2A_R. Ecto-5′-nucleotidase/CD73 is the rate limiting enzyme for ADO production from released adenine nucleotides, which plays a strategic role in calibrating the duration and magnitude of the purinergic signal delivered to immune cells [32] and to the motor endplate [11]. The study of adenosinergic-based therapies acting on A2AR and CD73 urges in order to provide novel strategies oriented simultaenously to suppress immune responses and to promote neuromuscular transmission. In this work we gathered information of the immunological and neuronal imbalance related to the adenosinergic pathway in a rat model of EAMG (see Figure 7), which compares to the human MG in both pathophysiological and clinical features.

To our knowledge, this is the first attempt to tackle common deficits affecting unbalanced neuromuscular transmission and autoimmune responses in a EAMG model. Insufficient amounts of adenosine to promote immune cells communication and neuromuscular transmission via A_2A_R activation seem to be operating in EAMG animals, yet the underlying mechanisms responsible for these findings seem to be diverse in the two systems. Deficits of ADO generation on immune cells are mainly dictated by a decreased expression of the ADO generating enzyme, ecto-5′-nucleotidase/CD73, on T_reg_ cells, which may be further aggravated by a decreased expression of A_2A_R on B and T cells [8]. The coordination, between increased serum ADA activities (Figure 1(a)) with the inability of T_reg_ cells to maintain high extracellular levels of ADO coming from adenine nucleotides (Figure 2), relieves suppression of specific nAChR-T lymphocytes leading to cell proliferation and to antigen-specific B cells activation in myasthenic animals (Figure 7). Regarding the motor endplate the lack of A_2A_R activity does not seem to be related to changes in the ability of ecto-5′-nucleotidase/CD73 to metabolize adenine nucleotides leading to ADO formation (Figure 6(a)) or to deficits in the A_2A_R receptor expression on motor nerve terminals (Figure 4(a)). The autoantibody attack on postsynaptic nAChR may compromise predominantly the retrograde release of ADO (Figure 5(a)) and/or its precursor, ATP, from affected skeletal muscle fibers, leading to a functional loss of A_2A_R-mediated facilitation of ACh release and to neuromuscular transmission failure in EAMG rats.

Highly incapacitating neuromuscular transmission deficits associated with MG, namely, muscle weakness and fatigability, may result from an attack of the complement system on antibody-nAChR complexes bound to the motor endplate leading to its disorganization and nAChR loss [30]. In this work, we extended the mechanisms associated to neuromuscular transmission failure in EAMG by implicating deficits in the ADO pathway. Like that observed with the toxin-induced MG animal model [7], data presented in this study indicates that endogenous adenosine generated in myasthenic motor endplates during repetitive nerve firing (Figure 5(a)) may be insufficient to preserve transmitter release during repetitive neuronal firing via tonic activation of presynaptic facilitatory A_2A_R (Figure 4(c)). The conjunction of these findings with the data already presented in the bungarotoxin-induced MG rat model, where no evidence of significant damage of muscle integrity have been reported [7, 36] suggests that a decreased accumulation of adenosine leading to insufficient tonic activity of A_2A_R on motor endplates of myasthenic animals may be linked to muscle paralysis caused by the loss of nAChR, instead of the immune mediated disruption of endplate morphology. Muscle paralysis with *μ*-conotoxin GIIIB, a toxin that blocks muscle-specific voltage-gated Na^+^ channels without affecting neuronal function [37], decreased nerve-evoked ATP (~15%) and ADO (>90%) outflow [7]. Moderate-to-severe MG patients have impaired oxidative metabolism and a noticeable shift to glycolytic metabolism during exercise, which yields to higher-end Pi/ATP ratio and reduced levels of synaptic ADO levels [38]. Besides the fact that exocytosis of ATP may occur synchronously with ACh in a frequency-dependent manner [13, 39], the nucleotide may also be released by tetanic stimulation of skeletal myotubes from healthy animals, through pannexin-1 hemichannels, within a 15 s to 3 min time scale [40]. Taken together, these findings imply that ATP and the end product of the ectonucleotidase cascade, ADO, might be considered essential retrograde mediators between muscle activity and synaptic adaptations, a situation that may be deregulated under pathological conditions, like the EAMG. At this point, one cannot exclude deficiencies in the conversion of released adenine nucleotides into ADO (by ecto-NTPDases) upstream the ecto-5′-nucleotidase/CD73, which may concur to explain the lack of ADO tone regulating neurotransmitter release at the motor endplate. This hypothesis certainly deserves further studies in the near future.

Our results show for the first time that immune suppression and neuromuscular transmission deficits in EAMG animals may be rehabilitated by A_2A_R activation, which can be achieved by shortcutting ecto-NTPDases with exogenous AMP serving as an ADO precursor (see Figures 3 and 6). Thus, maintenance of ecto-5′-nucleotidase/CD73 activity may be crucial to define the pattern of extracellular ATP-derived ADO formation favoring A_2A_R activation in order to rehabilitate cell communication deficits in myasthenics. Li and collaborators have already showed that administration of the A_2A_R agonist, CGS21680C, 29 days post EAMG induction (therapeutic treatment) ameliorated disease severity and decreased the number of Th1 and Th2 cells while increasing the number of Treg cells [8], thus suggesting that targeting A_2A_R may have putative therapeutic applications in T cell-based autoimmune diseases. The potential significant side effects of chronic systemic use of drugs acting on A_2A_R, as well as the residual action of the A_2A_R ligands on other ADO receptor subtypes, pushed forward a new era of drugs designed for local augmentation of the nucleoside function dictated by the proximity of ecto-5′-nucleotidase/CD73 [41, 42]. In this context, ecto-5′-nucleotidase/CD73 may assume a pivotal role as a pharmacological target to fine tune A_2A_R activity. The time-space coincidence expression of both ecto-5′-nucleotidase/CD73 and A_2A_R receptors offers new appealing therapeutic targets for immunotherapy and neuromuscular transmission reinforcement of myasthenia with minimal side effects.

## Abbreviations

EAMG: Experimental autoimmune* Myasthenia Gravis*
MG: * Myasthenia Gravis*
nAChR: Nicotinic acetylcholine receptor
A_2A_R: Adenosine A_2A_ receptor
AMP: Adenosine monophosphate
ADO: Adenosine
ADA: Adenosine Deaminase
CFA: Complete Freund adjuvant
IFA: Incomplete Freund adjuvant
FoxP3: Forkhead transcription factor 3
T_reg_: Regulatory T cells
LN: Lymph nodes
TMR-*α*-BTX: Tetramethylrhodamine conjugated with *α*-bungarotoxin
PEG-ADA: Pegylated bovine ADA, rb, rabbit, and ms, mouse.
